# Cancer Risk in Pediatric-Onset Inflammatory Bowel Disease

**DOI:** 10.3389/fped.2020.00400

**Published:** 2020-07-17

**Authors:** Wael El-Matary, Charles N. Bernstein

**Affiliations:** ^1^Department of Pediatrics, University of Manitoba, Winnipeg, MB, Canada; ^2^Children's Hospital Research Institute of Manitoba, Winnipeg, MB, Canada; ^3^IBD Clinical and Research Center, University of Manitoba, Winnipeg, MB, Canada; ^4^Department of Internal Medicine, University of Manitoba, Winnipeg, MB, Canada

**Keywords:** cancer, colitis, Crohn, inflammatory bowel disease, IBD, malignancy, pediatric

## Abstract

Inflammatory bowel disease (IBD) is a chronic, immune-mediated, non-curable disease. The incidence of IBD appears to have risen over the last few decades especially in the pediatric age group. IBD usually presents with gastrointestinal symptoms, including abdominal pain, diarrhea, and bleeding per rectum but can also be associated with systemic symptoms such as weight loss, fatigue, joint and skin problems, and psychological comorbidities. One major complication is gastrointestinal and extra-intestinal malignancy. This review discusses literature that focuses on cancer risk of pediatric-onset IBD.

Inflammatory bowel diseases (IBD) are chronic, immune-mediated disorders typically subdivided into three main categories: Crohn disease (CD), ulcerative colitis (UC), and IBD-unclassified (IBD-U). IBD are non-curable with a typical course of remissions and relapses. IBD affects 1:140 Canadians and it is predicted to affect 1% of the Canadian population by 2030 (1, 2). The incidence of IBD in the pediatric age group especially, has risen over the last few decades (3). Persons with IBD are at risk for several complications and co-morbid conditions which are either related to the disease itself or available therapies. One of the most serious potential complications is intestinal or extra-intestinal malignancy. Gastrointestinal cancers include colorectal cancer (CRC), intestinal lymphoma, small bowel adenocarcinoma, anal cancer, and cholangiocarcinoma while extra-intestinal associated cancers including lymphoma, and skin cancers (3–5).

Up to 25% of all IBD cases present in the pediatric age group. As pediatric-onset IBD is characterized by more extensive and aggressive disease, longer disease duration and a higher need for immune suppression early on, long-term cancer risk is a major concern especially among parents of children with IBD (6).

## Why do Patients With Inflammatory Bowel Disease Have an Increased Cancer Risk?

Increased risk of cancer in IBD can be divided into 2 main subdivisions:

### Disease-Related Cancer Risk

Cancer associated with chronic inflammation is characterized by a loss of normal growth regulation secondary to a number of genetic mutations and epigenetic alterations in pivotal cancer-related regulatory genes (7, 8). The cancer stem cell model postulates that expansion of stem cells occurs in response to these mutations, resulting in cancer development. Inflammation may affect DNA methylation, microsatellite stability, and histone modification. Cyclo-oxygenase 2, which metabolizes arachidonic acid to prostaglandins, is highly expressed in inflamed tissues and affects cell proliferation, apoptosis, aneuploidy, and angiogenesis. Moreover, chronic inflammation leads to production of reactive oxygen species and exaggerated cytokine and chemokine expression, such as tumor necrosis factor (TNF), interleukin (IL) 1, IL6, IL12, IL13, IL17, IL22, and IL23, by immune cells, which increase the risk of mutagenesis, genomic instability, and interactions between cancer stem cells and the local tumor microenvironment, including immune cells and myofibroblasts (7–12). Colonic inflammation may alter the sequence of genomic changes with subsequent widespread, multifocal, and augmented carcinogenesis (12).

Tumor suppressor protein p53 was found to be altered in up to 85% of colitis-associated cancers (13). Even in the absence of documented colonic cancer, up to 50% of biopsy samples taken from inflamed mucosa in patients with UC may have abnormal p53 indicating the significant role of inflammation in these mutations (13, 14).

Changes in intestinal microbiome may also contribute to cancer development as suggested by the difference in microbiome between those with CRC as compared to those without (15). Stool samples from patients with CRC had higher levels of certain bacteria such as *Enterococcus, Shigella, Firmicutes*, and *Bacteroidetes* with significantly lower levels of *Lachnospiraceae* as compared to controls without CRC. Animal studies have also shown regression in cancer development in antibiotic-treated mice. However, it is not clear yet how changing the microbiome could mediate or suppress carcinogenesis (15–18).

Similar mechanisms may be present in patients with CD as they are at a higher risk of developing ileal carcinoma (19).

The risk of CRC in pediatric-onset IBD is significantly increased as proven by the available evidence (Table 1). In a population-based case-control analysis, our group recently showed that 1.7% of patients with childhood-onset IBD developed cancer, as compared with 0.8% of controls [hazard ratio (HR) = 2.00, 95% confidence interval (CI) 1.16–3.43], with overall cancer rates of 114 and 57 per 100,000 person-years, respectively (4). Persons with CD had an HR for developing later cancers of 2.47 (95% CI 1.31–4.66), while in those with UC, the risk was numerically but not significantly increased (HR = 1.24, 95% CI 0.43–3.59). The study, however, reported all cancers as one group and it was not clear how many participants developed CRC (4).

**Table 1:** Summary of the main studies included in the review.

**Study**	**Location**	**Setting-design**	**Follow-up period**	**Main findings**
El-Matary (4)	Canada	Population-based with age, sex and location matched controls reporting any cancer	Persons with IBD and their controls were followed for 14,938 and 132,202 persons-years, respectively	1.7% of childhood-onset IBD developed cancer, as compared with 0.8% of controls HR = 2.00, (95% CI 1.16–3.43), with cancer rates of 114 and 57 per 100,000 person-years, respectively
El-Matary (20)	International (54 centers)	Retrospective hospital-based reporting CRC in children with PSC-UC	Median duration of follow up of 5 years	The incidence of colorectal dysplasia/cancer in pediatric PSC-UC was 2.8 cases per 1,000 person-years
Olen (21, 22)	Scandinavia	Population-based with age, sex, calendar year, and location matched controls reporting CRC in all age groups including pediatric onset IBD	The study period was from 1969 to 2017	CRC deaths in those diagnosed with UC before the age of 18 y to be 34.2 (95% CI 18.8–62.2). The risk was also elevated among those with CD
Olen (23)	Sweden	Cohort [national patient registry (excluding primary outpatient care)] with age, sex, and location matched controls reporting any cancer including CRC in pediatric-onset IBD	148,682 patient-years	HR for any cancer: UC: 2.6 (2.3–3.0) CD: 1.7 (1.5–2.1) HR for CRC UC: 33 (23-49) CD: 5.8 (3.2–10)
Joosse (24)	European multi-center	Retrospective hospital-based reporting any cancer in pediatric-onset IBD	Estimated 192,625 patient-years	Estimated cancer (any type) incidence of 171 per 1,000,000
Hyams (25)	North American multi-center	Prospective hospital-based reporting any cancer in pediatric-onset IBD	24,543 patient-years. A total follow up period of 9 year	15 cancers, eight of which were lymphoma and leukemia, and five hemophagocytic syndrome. Thiopurine exposed: 2.9 vs. Non-exposed: 1.3
Kappelman (26)	Denmark	Cohort using the Danish health care databases reporting any cancer in pediatric-onset IBD	Not clear	SIR for those with CD 2.3 and 2.0 for UC
Jess (27)	Denmark	Cohort (Nation-wide Cohort) reporting CRC in all age groups including pediatric-onset IBD (<20 y)	178 million person-years of follow-up	The relative risk for developing CRC in pediatric PSC-UC was 44 and 2 for CD. CRC did not increase except in those diagnosed with pediatric-onset UC or those with concomitant PSC
Peneau (28)	France	Cohort reporting any cancer in pediatric-onset IBD	Median follow up period of 15 years	Nine cancers including two with CRC SIR = 3.0 (1.3–5.9)
Ekbom (29)	Sweden	Population-based controlled study reporting CRC in pediatric-onset UC	4,220 person-years	SIR of CRC in patients diagnosed with UC (0–14 y) was 118.3 (95% CI 63–202.3)

*IBD, inflammatory bowel disease; UC, ulcerative colitis; CD, Crohn disease; CRC, colorectal cancer; PSC, primary sclerosing cholangitis; CI, confidence interval; HR, hazard ratio; SIR, standardized incidence ratio*.

In another population-based analysis, Ekbom et al. (29) reported the standardized incidence ratio (SIR) of CRC in patients diagnosed with UC (0–14 y) to be 118.3 (95% CI 63–202.3) times as compared to controls without IBD. Age at UC diagnosis was a strong independent prognostic factor for developing CRC i.e., the younger the age at UC diagnosis, the higher the risk for developing future CRC. The absolute risk of CRC 35 years after diagnosis of UC was 30% for patients with pancolitis at diagnosis and 40% in those diagnosed with UC before the age of 15 years (29).

In a large meta-analysis that examined the prevalence of CRC in patients with UC and included 116 studies, the overall prevalence of CRC in any patient with UC was 3.7% (95% CI 3.2–4.2%) (30). Only 41 studies reported the duration of colitis with an overall incidence rate of 3/1,000 person years. Of those, 18 studies reported the incidence of CRC in children with UC with only five having reported the duration of follow up. The overall incidence rate of CRC for any child with colitis was 6/1,000 person-years (95% CI 3/1,000–13/1,000). The cumulative probabilities of developing CRC were 5.5% within 10 years, 10.8% within 20 years, and 15.7% within 30 years following the diagnosis of UC (30).

In a large study based on a nation-wide registry, Olen et al. (23) reported an increased cancer risk in those diagnosed with IBD before the age of 18y. The study included 9,408 incident cases of IBD (4,648 with UC, 3,768 with CD, and 989 with IBD-U) compared with 92,870 age, sex, and location- matched controls. While there were only eight cancers (five lymphoid neoplasms, two CRC, and one small bowel cancer), the HR for any cancer was 2.6 in those with UC (95% CI 2.3–3.0) and 1.7 in CD (95% CI 1.5–2.1) compared to the general population (23). The HR for CRC in those with UC was 33 (95% CI 23–49) and 5.8 (95% CI 3.2–10) in those with CD. Similar to our study, persons with pediatric-onset IBD were at a 2-fold risk for developing any future cancer (2.2, 95% CI 2.0–2.5). Relative risks were increased in the first year after diagnosis and remained statistically significantly higher at 5 years of follow-up and onwards (23). A more recent study from the same group reported the adjusted HR for incident colorectal cancer deaths in those diagnosed with UC before the age of 18 y to be 34.2 (95% CI 18.8–62.2) (21). The risk was also elevated among those with CD (22).

In a population-based analysis that examined cancer risk in persons with IBD from a nationwide historical cohort using the Danish health care databases, the relative risk for invasive cancer one or more years after IBD diagnosis was highest for those diagnosed with IBD before 20 years of age (SIR for CD 2.3, 95% CI 1.5–3.4 and SIR for UC 2.0, 95% CI 1.4–1.7) (26). The details of cancers in the pediatric-onset IBD were not reported. On the other hand, and in another population-based study also from Denmark, the risk of CRC did not seem to be increased among persons with IBD except for those diagnosed with pediatric-onset UC or those with concomitant primary sclerosing cholangitis (PSC) (27). In those who were diagnosed with UC younger than age of 20 y, the relative risk for developing CRC was 44 (95% CI 27–719) (27).

The French EPIMAD registry reported the cancer incidence in their pediatric-onset IBD cohort (diagnosis of IBD <17 y of age) to be 1.3% over a median of follow up of 15 years. Of the nine reported cancers, only two were diagnosed as CRC but there was a significantly increased risk of cancer regardless of sex and age [SIR = 3.0 (1.3–5.9)] (28).

Another pediatric multi-center study from Europe reported 12 CRC in pediatric-onset IBD (8 UC/IBD-U and eight with isolated colonic CD) after a median duration of 9.3 years since IBD diagnosis. The estimated at-risk population of pediatric-onset IBD in Europe was 192,625 patient-years with an estimated cancer (any type) incidence of 171 per 1,000,000 which surprisingly was not significantly different from the reported cancer incidence in the European general population. Nonetheless, the study was limited by its design as it was neither a population-based nor registry based study suggesting a potential high probability of under-reporting (24).

We recently reported the incidence of colorectal dysplasia/cancer in pediatric PSC-UC to be 2.8 cases per 1,000 person-year. Fifty percentage of those who developed CRC were diagnosed with UC before the age of 6 years. The longer duration of inflammation may explain this finding. However, very-early-onset IBD could be an independent risk factor for developing CRC/dysplasia (20).

### Medication-Related Cancer Risk

Several medications are currently used to control inflammation in patients with IBD. Immunomodulators such as thiopurines (for example azathioprine (AZA) and 6-mercaptopurine) and methotrexate (MTX) or biological medications such as anti-TNF agents, are commonly used in children with IBD, but have been associated with concerns regarding cancer risk (19–35).

Thiopurines and MTX promote the development of cancer through several mechanisms including possible activation of several oncogenes, reduction in physiologic immunosurveillance of malignant cells, direct alteration in DNA and impaired immune response of oncogenic viruses (25, 35–37).

A recent study from the DEVELOP clinical cohort that prospectively collected data from 5,766 children with IBD reported 15 cancers, eight of which were lymphoma and leukemia, and five hemophagocytic syndrome (HLH) over a total follow up period of 9 year (38). Except for 2, all those who developed cancers or HLH were exposed to thiopurines and 10 of those with cancers received a biological agent. There was no difference in cancer risk between those who were exposed to infliximab (IFX) as compared to those who were not (36). The study raised a concern that AZA was integral for the development of malignancy or HLH in pediatric patients with IBD. In the Manitoba population based study, we could not demonstrate a statistically significant cancer risk in those who were exposed to AZA or IFX vs. those who did not (4).

Other reported cancers in the DEVELOP cohort included adenocarcinoma of the parotid gland, basal cell carcinoma, malignant melanoma, renal cell carcinoma, cholangiocarcinoma, and mycosis fungoides (38).

The Manitoba study also reported non-melanoma skin cancer, lymphoma, leukemia, and urinary bladder cancers in our cohort (4). Concordant with these results, a single-center, retrospective study of 1,374 children and young adults with IBD followed for a maximum period of 29 years for the development of lymphoma, there was not a significantly increased risk of lymphoma in the subset of children treated with thiopurines (39). Only two patients developed lymphoma (one Hodgkin, and one anaplastic large cell), in 6,624 patient-years of follow-up (mean duration follow-up 4.8 years per patient). Both patients were males (ages 12 and 18 years at time of lymphoma diagnosis) and were receiving thiopurines but had not yet received biologics. The absolute incidence rate of lymphoma for patients who received thiopurines was 4.5 per 10,000 patient-years compared to the expected rate of 0.58 per 10,000 patient-years, with SIR of 7.51 (95% CI 0.74–41.98) (39). While the sample size might be the reason of statistical insignificance, the absolute risk was still very low.

In 2008, The Food and Drug Administration (FDA) released investigatory reports of malignancy in children receiving anti-TNF agents for immune-related diseases including IBD (40). Thirty-one cases of malignancy in children receiving IFX were reported and 24 of them had IBD. Fatal hepatosplenic T cell lymphoma accounted for nine (all IBD) cases, followed by five cases with non-Hodgkin's lymphoma (NHL), three with Hodgkin's lymphoma, and three with leukemia. Other reported cancers included malignant melanoma, basal cell carcinoma, leiomyosarcoma, nephroblastoma, renal cell carcinoma, liver cancer, metastatic hepatocellular cancer, thyroid cancer, malignant mastocytosis, neuroblastoma, and colorectal cancer (one case each) (38). The median age of diagnosis was 17 y (65% were males) and IFX was started after a median time of 30 months from diagnosis. The majority of patients with IBD were receiving concomitant thiopurines and 4 of 24 were on concomitant MTX (40, 41). The report also included a 20-year old male who developed fatal hepatosplenic T cell lymphoma 8 months following the start of adalimumab for UC. Nonetheless, this patient previously received IFX and AZA before switching to adalimumab.

Since this report, there was a wide shift among pediatric gastroenterologists especially in North America from using AZA to MTX. Moreover, and because of the possible link of thiopurines to HLH, many gastroenterologists started screening their patients for Epstein Barr virus (EBV) antibodies before starting thiopurines. Interestingly, the majority of them would still start thiopurines in EBV-naïve patients (42, 43). Whether these strategies will reduce those complications or not is yet to be determined (44, 45).

## Conclusions

Pediatric patients with IBD are at increased risk of gastrointestinal and extra-intestinal malignancies, such as lymphomas, although the absolute risk is small. There is debate over whether lymphoma reported in IBD is a result of the patient's underlying disease, its treatment, or both (46). As the risk of CRC in pediatric-onset IBD is comparably high as that seen in adult-onset IBD, surveillance colonoscopy should probably start earlier and be performed at shorter intervals than currently recommended especially in high risk groups such as those with very early onset IBD. Currently very early-onset, pediatric-onset and adult-onset IBD are receiving the same surveillance strategy. The increased incidence of CRC in the young is a justification for revisiting the current guidelines for screening colonoscopy. However, further research is needed to define the optimal intervals for surveillance colonoscopy in these patients. Pediatric endoscopists need to be aware of this high risk and ensure adequacy in training for performing surveillance colonoscopy.

## Author Contributions

WE-M: conceptualization, interpretation of data, and writing original draft. CB: administration and reviewing and editing of manuscript. All authors contributed to the article and approved the submitted version.

## Conflict of Interest

WE-M served as an advisory board member for Janssen Canada and AbbVie Canada. He received a research support from Janssen. CB was supported in part by the Bigham Chair in Gastroenterology. He has consulted to Abbvie, Canada, Ferring Canada, Janssen Canada, Napo Pharmaceuticals, Pfizer Canada, Shire Canada, Takeda, Canada, and has consulted to Mylan Pharmaceuticals. He has received unrestricted educational grants from Abbvie Canada, Janssen Canada, Shire Canada, and Takeda Canada. He has been on speaker's bureau of Abbvie Canada, Ferring Canada and Shire Canada.
